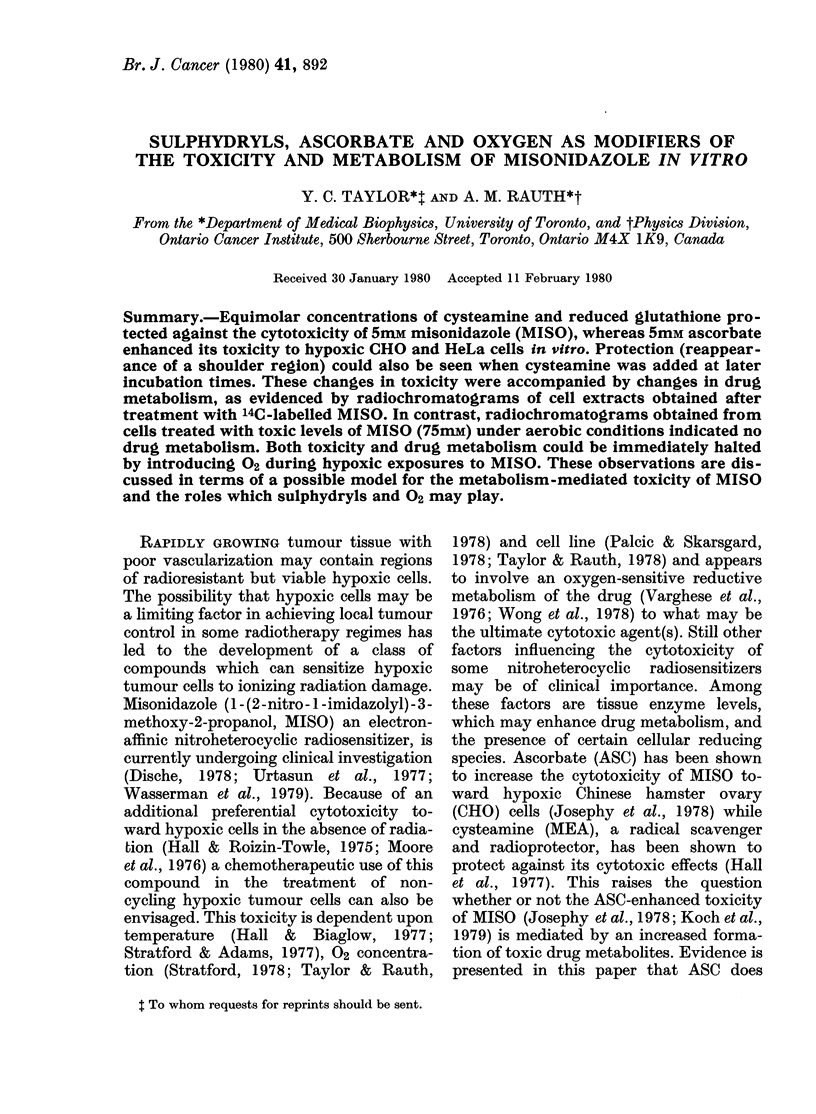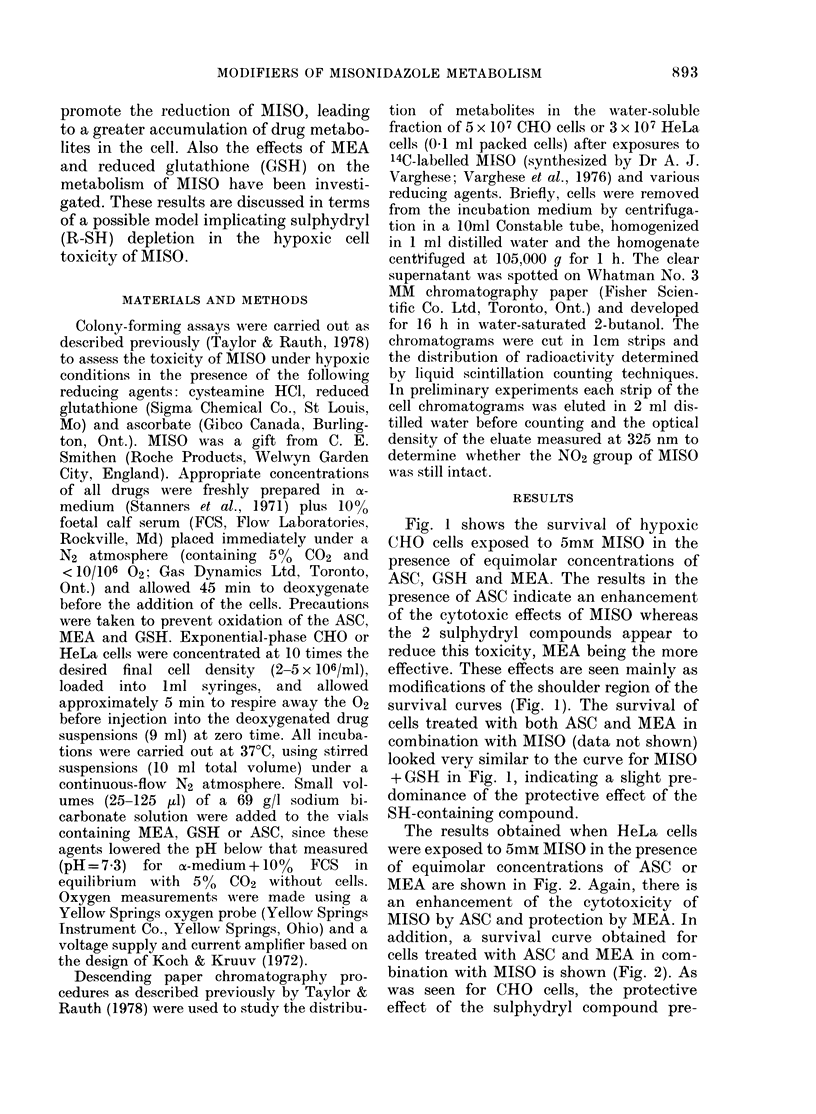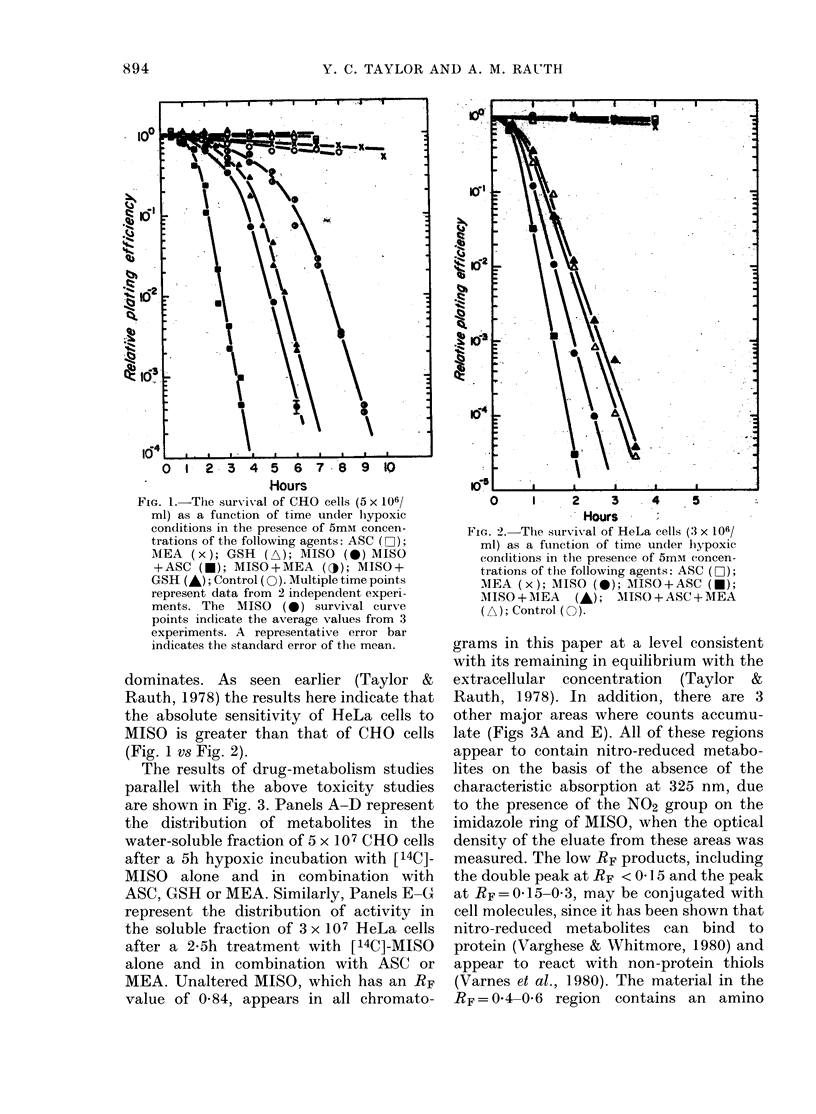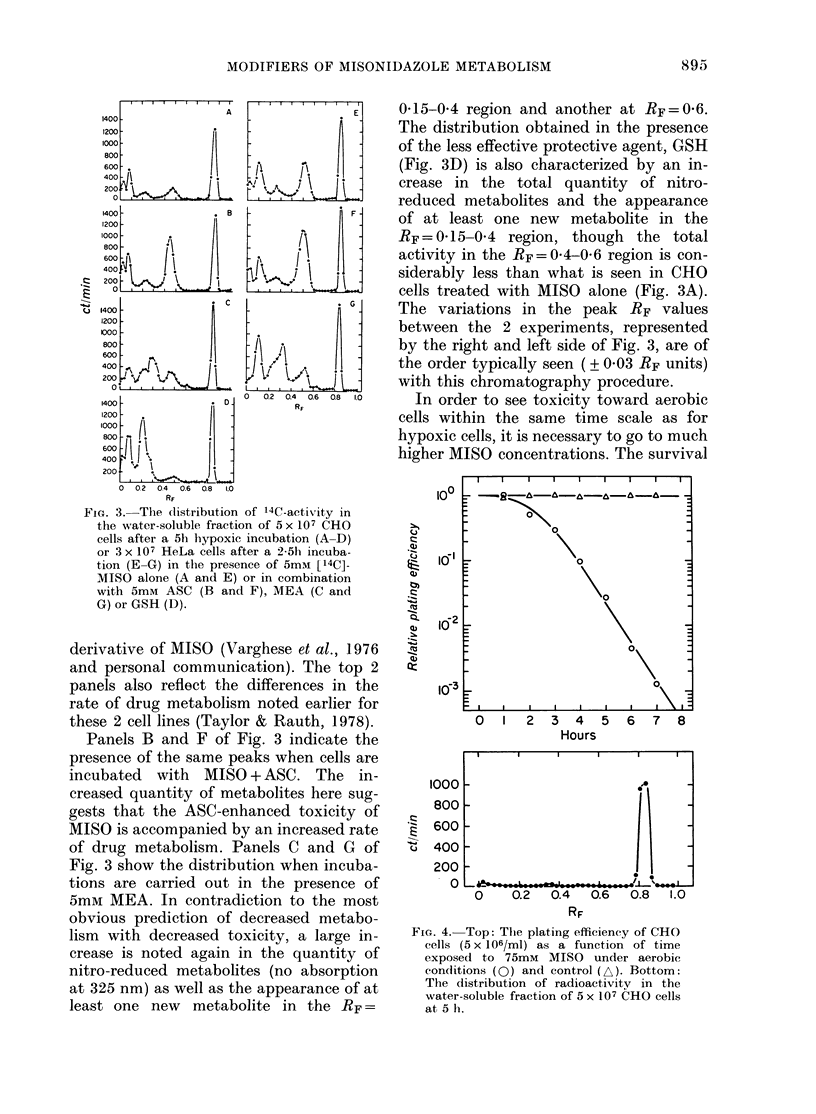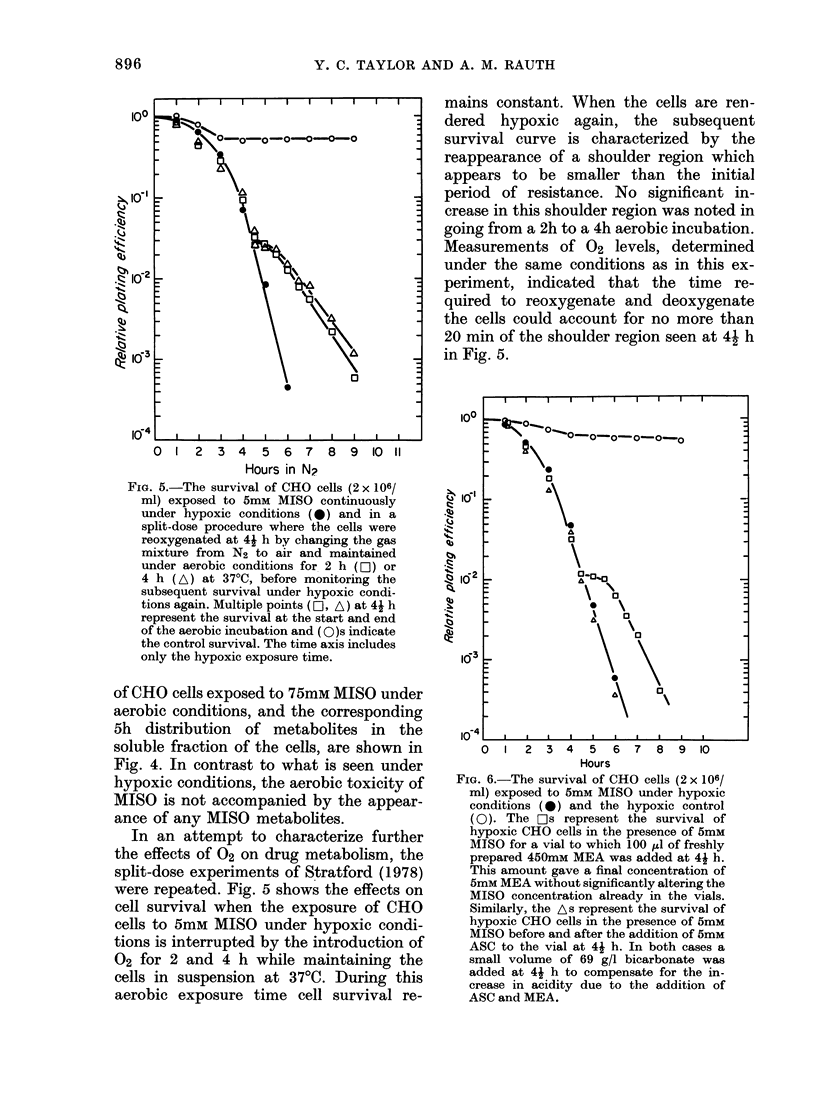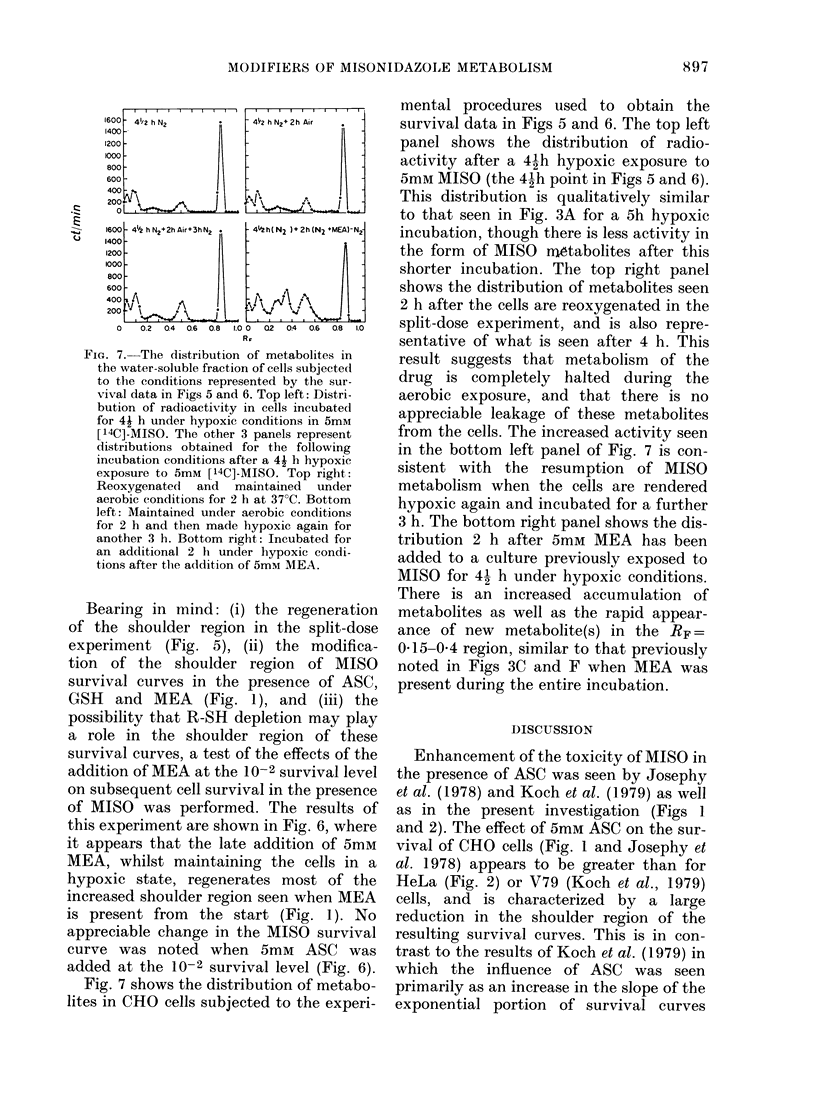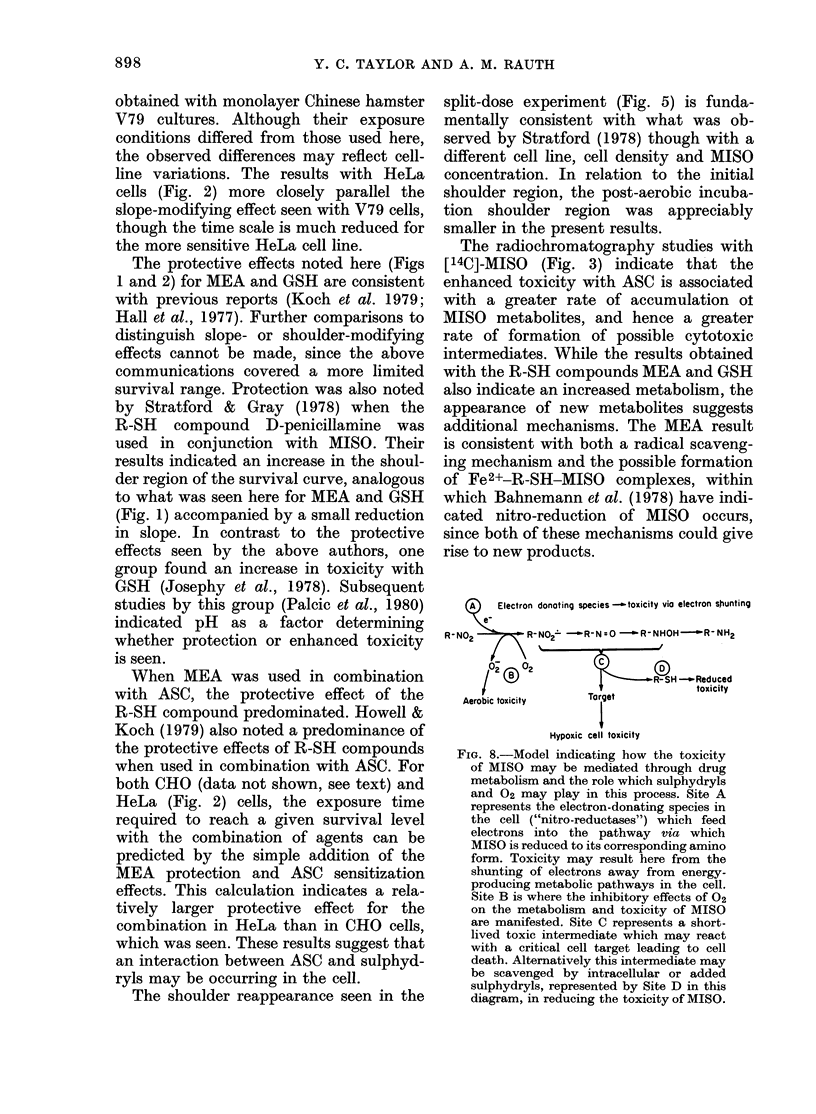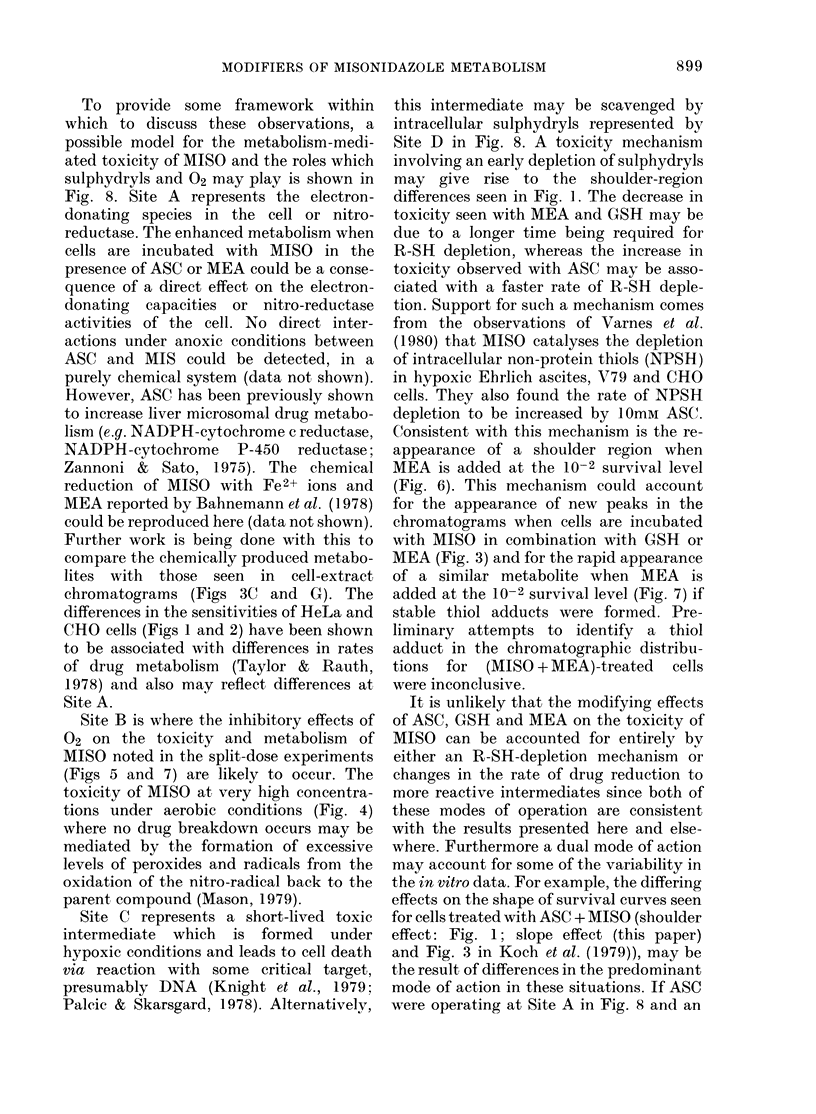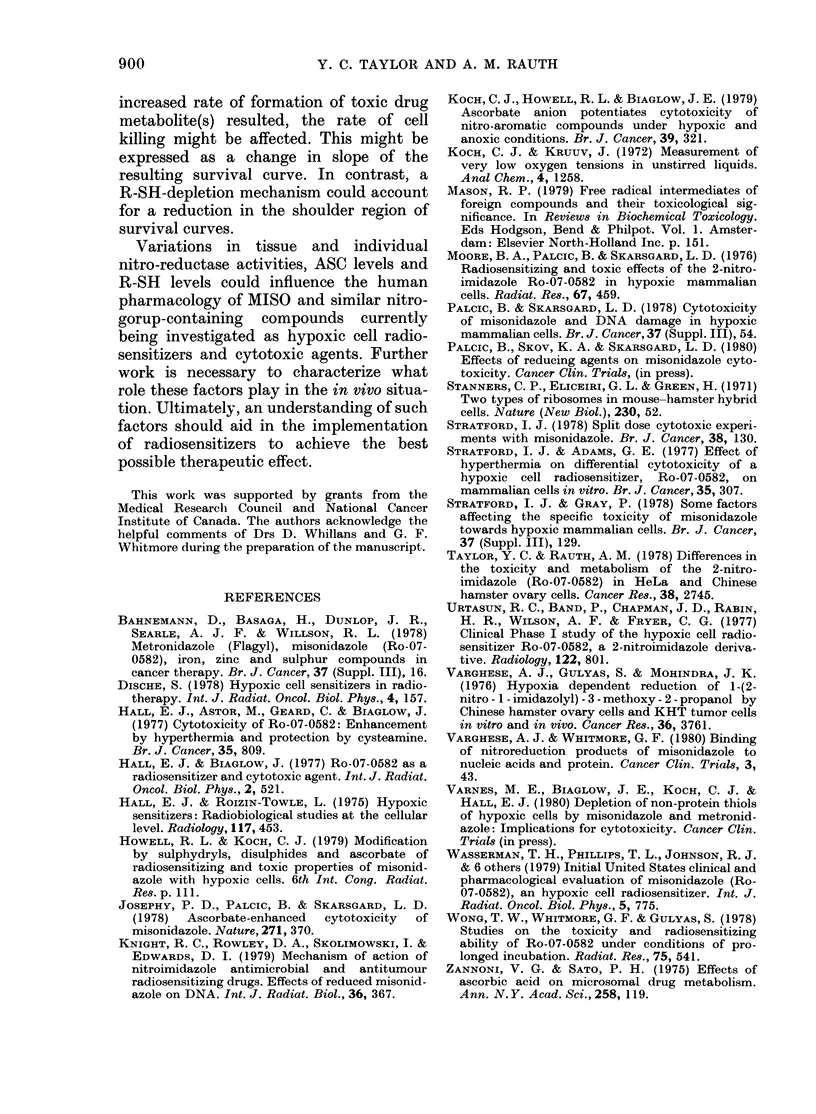# Sulphydryls, ascorbate and oxygen as modifiers of the toxicity and metabolism of misonidazole in vitro.

**DOI:** 10.1038/bjc.1980.166

**Published:** 1980-06

**Authors:** Y. C. Taylor, A. M. Rauth

## Abstract

Equimolar concentrations of cysteamine and reduced glutathione protected against the cytotoxicity of 5 mM misonidazole (MISO), whereas 5mM ascorbate enhanced its toxicity to hypoxic CHO and HeLa cells in vitro. Protection (reappearance of a shoulder region) could also be seen when cysteamine was added at later incubation times. These changes in toxicity were accompanied by changes in drug metabolism, as evidenced by radiochromatograms of cell extracts obtained after treatment with 14C-labelled MISO. In contrast, radiochromatograms obtained from cells treated with toxic levels of MISO (75 mM) under aerobic conditions indicated no drug metabolism. Both toxicity and drug metabolism could be immediately halted by introducing O2 during hypoxic exposure to MISO. These observations are discussed in terms of a possible model for the metabolism-mediated toxicity of MISO and the roles which sulphydryls and O2 may play.


*Br. J. Cancer* (1980) **41**, 892

### SULPHYDRYLS, ASCORBATE AND OXYGEN AS MODIFIERS OF THE TOXICITY AND METABOLISM OF MISONIDAZOLE *IN VITRO*

Y. C. TAYLOR*‡ AND A. M. RAUTH*†

*From the *Department of Medical Biophysics, University of Toronto, and †Physics Division, Ontario Cancer Institute, 500 Sherbourne Street, Toronto, Ontario M4X 1K9, Canada*

Received 30 January 1980 Accepted 11 February 1980

**Summary.—Equimolar concentrations of cysteamine and reduced glutathione protected against the cytotoxicity of 5mM misonidazole (MISO), whereas 5mM ascorbate enhanced its toxicity to hypoxic CHO and HeLa cells *in vitro*. Protection (reappearance of a shoulder region) could also be seen when cysteamine was added at later incubation times. These changes in toxicity were accompanied by changes in drug metabolism, as evidenced by radiochromatograms of cell extracts obtained after treatment with $^{14}C$-labelled MISO. In contrast, radiochromatograms obtained from cells treated with toxic levels of MISO (75mM) under aerobic conditions indicated no drug metabolism. Both toxicity and drug metabolism could be immediately halted by introducing $O_2$ during hypoxic exposures to MISO. These observations are discussed in terms of a possible model for the metabolism-mediated toxicity of MISO and the roles which sulphydryls and $O_2$ may play.**

RAPIDLY GROWING tumour tissue with poor vascularization may contain regions of radioresistant but viable hypoxic cells. The possibility that hypoxic cells may be a limiting factor in achieving local tumour control in some radiotherapy regimes has led to the development of a class of compounds which can sensitize hypoxic tumour cells to ionizing radiation damage. Misonidazole (1-(2-nitro-1-imidazolyl)-3-methoxy-2-propanol, MISO) an electron-affinic nitroheterocyclic radiosensitizer, is currently undergoing clinical investigation (Dische, 1978; Urtasun *et al.*, 1977; Wasserman *et al.*, 1979). Because of an additional preferential cytotoxicity toward hypoxic cells in the absence of radiation (Hall & Roizin-Towle, 1975; Moore *et al.*, 1976) a chemotherapeutic use of this compound in the treatment of non-cycling hypoxic tumour cells can also be envisaged. This toxicity is dependent upon temperature (Hall & Biaglow, 1977; Stratford & Adams, 1977), $O_2$ concentration (Stratford, 1978; Taylor & Rauth, 1978) and cell line (Palcic & Skarsgard, 1978; Taylor & Rauth, 1978) and appears to involve an oxygen-sensitive reductive metabolism of the drug (Varghese *et al.*, 1976; Wong *et al.*, 1978) to what may be the ultimate cytotoxic agent(s). Still other factors influencing the cytotoxicity of some nitroheterocyclic radiosensitizers may be of clinical importance. Among these factors are tissue enzyme levels, which may enhance drug metabolism, and the presence of certain cellular reducing species. Ascorbate (ASC) has been shown to increase the cytotoxicity of MISO toward hypoxic Chinese hamster ovary (CHO) cells (Josephy *et al.*, 1978) while cysteamine (MEA), a radical scavenger and radioprotector, has been shown to protect against its cytotoxic effects (Hall *et al.*, 1977). This raises the question whether or not the ASC-enhanced toxicity of MISO (Josephy *et al.*, 1978; Koch *et al.*, 1979) is mediated by an increased formation of toxic drug metabolites. Evidence is presented in this paper that ASC does

‡ To whom requests for reprints should be sent.

promote the reduction of MISO, leading to a greater accumulation of drug metabolites in the cell. Also the effects of MEA and reduced glutathione (GSH) on the metabolism of MISO have been investigated. These results are discussed in terms of a possible model implicating sulphydryl (R-SH) depletion in the hypoxic cell toxicity of MISO.

### MATERIALS AND METHODS

Colony-forming assays were carried out as described previously (Taylor & Rauth, 1978) to assess the toxicity of MISO under hypoxic conditions in the presence of the following reducing agents: cysteamine HCl, reduced glutathione (Sigma Chemical Co., St Louis, Mo) and ascorbate (Gibco Canada, Burlington, Ont.). MISO was a gift from C. E. Smithen (Roche Products, Welwyn Garden City, England). Appropriate concentrations of all drugs were freshly prepared in α-medium (Stanners *et al.*, 1971) plus 10% foetal calf serum (FCS, Flow Laboratories, Rockville, Md) placed immediately under a $N_2$ atmosphere (containing 5% $CO_2$ and $<10/10^6$ $O_2$; Gas Dynamics Ltd, Toronto, Ont.) and allowed 45 min to deoxygenate before the addition of the cells. Precautions were taken to prevent oxidation of the ASC, MEA and GSH. Exponential-phase CHO or HeLa cells were concentrated at 10 times the desired final cell density ($2–5\times10^6$/ml), loaded into 1ml syringes, and allowed approximately 5 min to respire away the $O_2$ before injection into the deoxygenated drug suspensions (9 ml) at zero time. All incubations were carried out at 37°C, using stirred suspensions (10 ml total volume) under a continuous-flow $N_2$ atmosphere. Small volumes (25–125 μl) of a 69 g/l sodium bicarbonate solution were added to the vials containing MEA, GSH or ASC, since these agents lowered the pH below that measured (pH = 7·3) for α-medium + 10% FCS in equilibrium with 5% $CO_2$ without cells. Oxygen measurements were made using a Yellow Springs oxygen probe (Yellow Springs Instrument Co., Yellow Springs, Ohio) and a voltage supply and current amplifier based on the design of Koch & Kruuv (1972).

Descending paper chromatography procedures as described previously by Taylor & Rauth (1978) were used to study the distribution of metabolites in the water-soluble fraction of $5\times10^7$ CHO cells or $3\times10^7$ HeLa cells (0·1 ml packed cells) after exposures to $^{14}$C-labelled MISO (synthesized by Dr A. J. Varghese; Varghese *et al.*, 1976) and various reducing agents. Briefly, cells were removed from the incubation medium by centrifugation in a 10ml Constable tube, homogenized in 1 ml distilled water and the homogenate centrifuged at 105,000 *g* for 1 h. The clear supernatant was spotted on Whatman No. 3 MM chromatography paper (Fisher Scientific Co. Ltd, Toronto, Ont.) and developed for 16 h in water-saturated 2-butanol. The chromatograms were cut in 1cm strips and the distribution of radioactivity determined by liquid scintillation counting techniques. In preliminary experiments each strip of the cell chromatograms was eluted in 2 ml distilled water before counting and the optical density of the eluate measured at 325 nm to determine whether the $NO_2$ group of MISO was still intact.

### RESULTS

Fig. 1 shows the survival of hypoxic CHO cells exposed to 5mM MISO in the presence of equimolar concentrations of ASC, GSH and MEA. The results in the presence of ASC indicate an enhancement of the cytotoxic effects of MISO whereas the 2 sulphydryl compounds appear to reduce this toxicity, MEA being the more effective. These effects are seen mainly as modifications of the shoulder region of the survival curves (Fig. 1). The survival of cells treated with both ASC and MEA in combination with MISO (data not shown) looked very similar to the curve for MISO + GSH in Fig. 1, indicating a slight predominance of the protective effect of the SH-containing compound.

The results obtained when HeLa cells were exposed to 5mM MISO in the presence of equimolar concentrations of ASC or MEA are shown in Fig. 2. Again, there is an enhancement of the cytotoxicity of MISO by ASC and protection by MEA. In addition, a survival curve obtained for cells treated with ASC and MEA in combination with MISO is shown (Fig. 2). As was seen for CHO cells, the protective effect of the sulphydryl compound pre-

FIG. 1.—The survival of CHO cells ($5 \times 10^6$/ml) as a function of time under hypoxic conditions in the presence of 5mM concentrations of the following agents: ASC (□); MEA (×); GSH (△); MISO (●) MISO +ASC (■); MISO+MEA (◑); MISO+GSH (▲); Control (○). Multiple time points represent data from 2 independent experiments. The MISO (●) survival curve points indicate the average values from 3 experiments. A representative error bar indicates the standard error of the mean.

FIG. 2.—The survival of HeLa cells ($3 \times 10^6$/ml) as a function of time under hypoxic conditions in the presence of 5mM concentrations of the following agents: ASC (□); MEA (×); MISO (●); MISO+ASC (■); MISO+MEA (▲); MISO+ASC+MEA (△); Control (○).

dominates. As seen earlier (Taylor & Rauth, 1978) the results here indicate that the absolute sensitivity of HeLa cells to MISO is greater than that of CHO cells (Fig. 1 *vs* Fig. 2).

The results of drug-metabolism studies parallel with the above toxicity studies are shown in Fig. 3. Panels A–D represent the distribution of metabolites in the water-soluble fraction of $5 \times 10^7$ CHO cells after a 5h hypoxic incubation with [$^{14}$C]-MISO alone and in combination with ASC, GSH or MEA. Similarly, Panels E–G represent the distribution of activity in the soluble fraction of $3 \times 10^7$ HeLa cells after a 2·5h treatment with [$^{14}$C]-MISO alone and in combination with ASC or MEA. Unaltered MISO, which has an $R_F$ value of 0·84, appears in all chromatograms in this paper at a level consistent with its remaining in equilibrium with the extracellular concentration (Taylor & Rauth, 1978). In addition, there are 3 other major areas where counts accumulate (Figs 3A and E). All of these regions appear to contain nitro-reduced metabolites on the basis of the absence of the characteristic absorption at 325 nm, due to the presence of the $NO_2$ group on the imidazole ring of MISO, when the optical density of the eluate from these areas was measured. The low $R_F$ products, including the double peak at $R_F < 0{\cdot}15$ and the peak at $R_F = 0{\cdot}15$–$0{\cdot}3$, may be conjugated with cell molecules, since it has been shown that nitro-reduced metabolites can bind to protein (Varghese & Whitmore, 1980) and appear to react with non-protein thiols (Varnes *et al.*, 1980). The material in the $R_F = 0{\cdot}4$–$0{\cdot}6$ region contains an amino

FIG. 3.—The distribution of $^{14}C$-activity in the water-soluble fraction of $5 \times 10^7$ CHO cells after a 5h hypoxic incubation (A–D) or $3 \times 10^7$ HeLa cells after a 2·5h incubation (E–G) in the presence of 5mM [$^{14}C$]-MISO alone (A and E) or in combination with 5mM ASC (B and F), MEA (C and G) or GSH (D).

derivative of MISO (Varghese *et al.*, 1976 and personal communication). The top 2 panels also reflect the differences in the rate of drug metabolism noted earlier for these 2 cell lines (Taylor & Rauth, 1978).

Panels B and F of Fig. 3 indicate the presence of the same peaks when cells are incubated with MISO + ASC. The increased quantity of metabolites here suggests that the ASC-enhanced toxicity of MISO is accompanied by an increased rate of drug metabolism. Panels C and G of Fig. 3 show the distribution when incubations are carried out in the presence of 5mM MEA. In contradiction to the most obvious prediction of decreased metabolism with decreased toxicity, a large increase is noted again in the quantity of nitro-reduced metabolites (no absorption at 325 nm) as well as the appearance of at least one new metabolite in the $R_F$ = 0·15–0·4 region and another at $R_F = 0·6$. The distribution obtained in the presence of the less effective protective agent, GSH (Fig. 3D) is also characterized by an increase in the total quantity of nitro-reduced metabolites and the appearance of at least one new metabolite in the $R_F = 0·15–0·4$ region, though the total activity in the $R_F = 0·4–0·6$ region is considerably less than what is seen in CHO cells treated with MISO alone (Fig. 3A). The variations in the peak $R_F$ values between the 2 experiments, represented by the right and left side of Fig. 3, are of the order typically seen ($\pm$ 0·03 $R_F$ units) with this chromatography procedure.

In order to see toxicity toward aerobic cells within the same time scale as for hypoxic cells, it is necessary to go to much higher MISO concentrations. The survival

FIG. 4.—Top: The plating efficiency of CHO cells ($5 \times 10^6$/ml) as a function of time exposed to 75mM MISO under aerobic conditions (○) and control (△). Bottom: The distribution of radioactivity in the water-soluble fraction of $5 \times 10^7$ CHO cells at 5 h.

FIG. 5.—The survival of CHO cells ($2 \times 10^6$/ml) exposed to 5mM MISO continuously under hypoxic conditions (●) and in a split-dose procedure where the cells were reoxygenated at $4\frac{1}{2}$ h by changing the gas mixture from $N_2$ to air and maintained under aerobic conditions for 2 h (□) or 4 h (△) at 37°C, before monitoring the subsequent survival under hypoxic conditions again. Multiple points (□, △) at $4\frac{1}{2}$ h represent the survival at the start and end of the aerobic incubation and (○)s indicate the control survival. The time axis includes only the hypoxic exposure time.

of CHO cells exposed to 75mM MISO under aerobic conditions, and the corresponding 5h distribution of metabolites in the soluble fraction of the cells, are shown in Fig. 4. In contrast to what is seen under hypoxic conditions, the aerobic toxicity of MISO is not accompanied by the appearance of any MISO metabolites.

In an attempt to characterize further the effects of $O_2$ on drug metabolism, the split-dose experiments of Stratford (1978) were repeated. Fig. 5 shows the effects on cell survival when the exposure of CHO cells to 5mM MISO under hypoxic conditions is interrupted by the introduction of $O_2$ for 2 and 4 h while maintaining the cells in suspension at 37°C. During this aerobic exposure time cell survival remains constant. When the cells are rendered hypoxic again, the subsequent survival curve is characterized by the reappearance of a shoulder region which appears to be smaller than the initial period of resistance. No significant increase in this shoulder region was noted in going from a 2h to a 4h aerobic incubation. Measurements of $O_2$ levels, determined under the same conditions as in this experiment, indicated that the time required to reoxygenate and deoxygenate the cells could account for no more than 20 min of the shoulder region seen at $4\frac{1}{2}$ h in Fig. 5.

FIG. 6.—The survival of CHO cells ($2 \times 10^6$/ml) exposed to 5mM MISO under hypoxic conditions (●) and the hypoxic control (○). The □s represent the survival of hypoxic CHO cells in the presence of 5mM MISO for a vial to which 100 μl of freshly prepared 450mM MEA was added at $4\frac{1}{2}$ h. This amount gave a final concentration of 5mM MEA without significantly altering the MISO concentration already in the vials. Similarly, the △s represent the survival of hypoxic CHO cells in the presence of 5mM MISO before and after the addition of 5mM ASC to the vial at $4\frac{1}{2}$ h. In both cases a small volume of 69 g/l bicarbonate was added at $4\frac{1}{2}$ h to compensate for the increase in acidity due to the addition of ASC and MEA.

FIG. 7.—The distribution of metabolites in the water-soluble fraction of cells subjected to the conditions represented by the survival data in Figs 5 and 6. Top left: Distribution of radioactivity in cells incubated for 4½ h under hypoxic conditions in 5mM [$^{14}$C]-MISO. The other 3 panels represent distributions obtained for the following incubation conditions after a 4½ h hypoxic exposure to 5mM [$^{14}$C]-MISO. Top right: Reoxygenated and maintained under aerobic conditions for 2 h at 37°C. Bottom left: Maintained under aerobic conditions for 2 h and then made hypoxic again for another 3 h. Bottom right: Incubated for an additional 2 h under hypoxic conditions after the addition of 5mM MEA.

Bearing in mind: (i) the regeneration of the shoulder region in the split-dose experiment (Fig. 5), (ii) the modification of the shoulder region of MISO survival curves in the presence of ASC, GSH and MEA (Fig. 1), and (iii) the possibility that R-SH depletion may play a role in the shoulder region of these survival curves, a test of the effects of the addition of MEA at the $10^{-2}$ survival level on subsequent cell survival in the presence of MISO was performed. The results of this experiment are shown in Fig. 6, where it appears that the late addition of 5mM MEA, whilst maintaining the cells in a hypoxic state, regenerates most of the increased shoulder region seen when MEA is present from the start (Fig. 1). No appreciable change in the MISO survival curve was noted when 5mM ASC was added at the $10^{-2}$ survival level (Fig. 6).

Fig. 7 shows the distribution of metabolites in CHO cells subjected to the experimental procedures used to obtain the survival data in Figs 5 and 6. The top left panel shows the distribution of radioactivity after a 4½h hypoxic exposure to 5mM MISO (the 4½h point in Figs 5 and 6). This distribution is qualitatively similar to that seen in Fig. 3A for a 5h hypoxic incubation, though there is less activity in the form of MISO metabolites after this shorter incubation. The top right panel shows the distribution of metabolites seen 2 h after the cells are reoxygenated in the split-dose experiment, and is also representative of what is seen after 4 h. This result suggests that metabolism of the drug is completely halted during the aerobic exposure, and that there is no appreciable leakage of these metabolites from the cells. The increased activity seen in the bottom left panel of Fig. 7 is consistent with the resumption of MISO metabolism when the cells are rendered hypoxic again and incubated for a further 3 h. The bottom right panel shows the distribution 2 h after 5mM MEA has been added to a culture previously exposed to MISO for 4½ h under hypoxic conditions. There is an increased accumulation of metabolites as well as the rapid appearance of new metabolite(s) in the $R_F$ = 0·15–0·4 region, similar to that previously noted in Figs 3C and F when MEA was present during the entire incubation.

### DISCUSSION

Enhancement of the toxicity of MISO in the presence of ASC was seen by Josephy *et al.* (1978) and Koch *et al.* (1979) as well as in the present investigation (Figs 1 and 2). The effect of 5mM ASC on the survival of CHO cells (Fig. 1 and Josephy *et al.* 1978) appears to be greater than for HeLa (Fig. 2) or V79 (Koch *et al.*, 1979) cells, and is characterized by a large reduction in the shoulder region of the resulting survival curves. This is in contrast to the results of Koch *et al.* (1979) in which the influence of ASC was seen primarily as an increase in the slope of the exponential portion of survival curves obtained with monolayer Chinese hamster V79 cultures. Although their exposure conditions differed from those used here, the observed differences may reflect cell-line variations. The results with HeLa cells (Fig. 2) more closely parallel the slope-modifying effect seen with V79 cells, though the time scale is much reduced for the more sensitive HeLa cell line.

The protective effects noted here (Figs 1 and 2) for MEA and GSH are consistent with previous reports (Koch *et al.* 1979; Hall *et al.*, 1977). Further comparisons to distinguish slope- or shoulder-modifying effects cannot be made, since the above communications covered a more limited survival range. Protection was also noted by Stratford & Gray (1978) when the R-SH compound D-penicillamine was used in conjunction with MISO. Their results indicated an increase in the shoulder region of the survival curve, analogous to what was seen here for MEA and GSH (Fig. 1) accompanied by a small reduction in slope. In contrast to the protective effects seen by the above authors, one group found an increase in toxicity with GSH (Josephy *et al.*, 1978). Subsequent studies by this group (Palcic *et al.*, 1980) indicated pH as a factor determining whether protection or enhanced toxicity is seen.

When MEA was used in combination with ASC, the protective effect of the R-SH compound predominated. Howell & Koch (1979) also noted a predominance of the protective effects of R-SH compounds when used in combination with ASC. For both CHO (data not shown, see text) and HeLa (Fig. 2) cells, the exposure time required to reach a given survival level with the combination of agents can be predicted by the simple addition of the MEA protection and ASC sensitization effects. This calculation indicates a relatively larger protective effect for the combination in HeLa than in CHO cells, which was seen. These results suggest that an interaction between ASC and sulphydryls may be occurring in the cell.

The shoulder reappearance seen in the split-dose experiment (Fig. 5) is fundamentally consistent with what was observed by Stratford (1978) though with a different cell line, cell density and MISO concentration. In relation to the initial shoulder region, the post-aerobic incubation shoulder region was appreciably smaller in the present results.

The radiochromatography studies with [$^{14}$C]-MISO (Fig. 3) indicate that the enhanced toxicity with ASC is associated with a greater rate of accumulation ot MISO metabolites, and hence a greater rate of formation of possible cytotoxic intermediates. While the results obtained with the R-SH compounds MEA and GSH also indicate an increased metabolism, the appearance of new metabolites suggests additional mechanisms. The MEA result is consistent with both a radical scavenging mechanism and the possible formation of $Fe^{2+}$–R-SH–MISO complexes, within which Bahnemann *et al.* (1978) have indicated nitro-reduction of MISO occurs, since both of these mechanisms could give rise to new products.

FIG. 8.—Model indicating how the toxicity of MISO may be mediated through drug metabolism and the role which sulphydryls and $O_2$ may play in this process. Site A represents the electron-donating species in the cell ("nitro-reductases") which feed electrons into the pathway *via* which MISO is reduced to its corresponding amino form. Toxicity may result here from the shunting of electrons away from energy-producing metabolic pathways in the cell. Site B is where the inhibitory effects of $O_2$ on the metabolism and toxicity of MISO are manifested. Site C represents a short-lived toxic intermediate which may react with a critical cell target leading to cell death. Alternatively this intermediate may be scavenged by intracellular or added sulphydryls, represented by Site D in this diagram, in reducing the toxicity of MISO.

To provide some framework within which to discuss these observations, a possible model for the metabolism-mediated toxicity of MISO and the roles which sulphydryls and $O_2$ may play is shown in Fig. 8. Site A represents the electron-donating species in the cell or nitro-reductase. The enhanced metabolism when cells are incubated with MISO in the presence of ASC or MEA could be a consequence of a direct effect on the electron-donating capacities or nitro-reductase activities of the cell. No direct interactions under anoxic conditions between ASC and MIS could be detected, in a purely chemical system (data not shown). However, ASC has been previously shown to increase liver microsomal drug metabolism (*e.g.* NADPH-cytochrome c reductase, NADPH-cytochrome P-450 reductase; Zannoni & Sato, 1975). The chemical reduction of MISO with $Fe^{2+}$ ions and MEA reported by Bahnemann *et al.* (1978) could be reproduced here (data not shown). Further work is being done with this to compare the chemically produced metabolites with those seen in cell-extract chromatograms (Figs 3C and G). The differences in the sensitivities of HeLa and CHO cells (Figs 1 and 2) have been shown to be associated with differences in rates of drug metabolism (Taylor & Rauth, 1978) and also may reflect differences at Site A.

Site B is where the inhibitory effects of $O_2$ on the toxicity and metabolism of MISO noted in the split-dose experiments (Figs 5 and 7) are likely to occur. The toxicity of MISO at very high concentrations under aerobic conditions (Fig. 4) where no drug breakdown occurs may be mediated by the formation of excessive levels of peroxides and radicals from the oxidation of the nitro-radical back to the parent compound (Mason, 1979).

Site C represents a short-lived toxic intermediate which is formed under hypoxic conditions and leads to cell death *via* reaction with some critical target, presumably DNA (Knight *et al.*, 1979; Palcic & Skarsgard, 1978). Alternatively, this intermediate may be scavenged by intracellular sulphydryls represented by Site D in Fig. 8. A toxicity mechanism involving an early depletion of sulphydryls may give rise to the shoulder-region differences seen in Fig. 1. The decrease in toxicity seen with MEA and GSH may be due to a longer time being required for R-SH depletion, whereas the increase in toxicity observed with ASC may be associated with a faster rate of R-SH depletion. Support for such a mechanism comes from the observations of Varnes *et al.* (1980) that MISO catalyses the depletion of intracellular non-protein thiols (NPSH) in hypoxic Ehrlich ascites, V79 and CHO cells. They also found the rate of NPSH depletion to be increased by 10mM ASC. Consistent with this mechanism is the reappearance of a shoulder region when MEA is added at the $10^{-2}$ survival level (Fig. 6). This mechanism could account for the appearance of new peaks in the chromatograms when cells are incubated with MISO in combination with GSH or MEA (Fig. 3) and for the rapid appearance of a similar metabolite when MEA is added at the $10^{-2}$ survival level (Fig. 7) if stable thiol adducts were formed. Preliminary attempts to identify a thiol adduct in the chromatographic distributions for (MISO + MEA)-treated cells were inconclusive.

It is unlikely that the modifying effects of ASC, GSH and MEA on the toxicity of MISO can be accounted for entirely by either an R-SH-depletion mechanism or changes in the rate of drug reduction to more reactive intermediates since both of these modes of operation are consistent with the results presented here and elsewhere. Furthermore a dual mode of action may account for some of the variability in the *in vitro* data. For example, the differing effects on the shape of survival curves seen for cells treated with ASC + MISO (shoulder effect: Fig. 1; slope effect (this paper) and Fig. 3 in Koch *et al.* (1979)), may be the result of differences in the predominant mode of action in these situations. If ASC were operating at Site A in Fig. 8 and an increased rate of formation of toxic drug metabolite(s) resulted, the rate of cell killing might be affected. This might be expressed as a change in slope of the resulting survival curve. In contrast, a R-SH-depletion mechanism could account for a reduction in the shoulder region of survival curves.

Variations in tissue and individual nitro-reductase activities, ASC levels and R-SH levels could influence the human pharmacology of MISO and similar nitro-gorup-containing compounds currently being investigated as hypoxic cell radiosensitizers and cytotoxic agents. Further work is necessary to characterize what role these factors play in the *in vivo* situation. Ultimately, an understanding of such factors should aid in the implementation of radiosensitizers to achieve the best possible therapeutic effect.

This work was supported by grants from the Medical Research Council and National Cancer Institute of Canada. The authors acknowledge the helpful comments of Drs D. Whillans and G. F. Whitmore during the preparation of the manuscript.

### REFERENCES

BAHNEMANN, D., BASAGA, H., DUNLOP, J. R., SEARLE, A. J. F. & WILLSON, R. L. (1978) Metronidazole (Flagyl), misonidazole (Ro-07-0582), iron, zinc and sulphur compounds in cancer therapy. *Br. J. Cancer*, **37** (Suppl. III), 16.

DISCHE, S. (1978) Hypoxic cell sensitizers in radiotherapy. *Int. J. Radiat. Oncol. Biol. Phys.*, **4**, 157.

HALL, E. J., ASTOR, M., GEARD, C. & BIAGLOW, J. (1977) Cytotoxicity of Ro-07-0582: Enhancement by hyperthermia and protection by cysteamine. *Br. J. Cancer*, **35**, 809.

HALL, E. J. & BIAGLOW, J. (1977) Ro-07-0582 as a radiosensitizer and cytotoxic agent. *Int. J. Radiat. Oncol. Biol. Phys.*, **2**, 521.

HALL, E. J. & ROIZIN-TOWLE, L. (1975) Hypoxic sensitizers: Radiobiological studies at the cellular level. *Radiology*, **117**, 453.

HOWELL, R. L. & KOCH, C. J. (1979) Modification by sulphydryls, disulphides and ascorbate of radiosensitizing and toxic properties of misonidazole with hypoxic cells. *6th Int. Cong. Radiat. Res.* p. 111.

JOSEPHY, P. D., PALCIC, B. & SKARSGARD, L. D. (1978) Ascorbate-enhanced cytotoxicity of misonidazole. *Nature*, **271**, 370.

KNIGHT, R. C., ROWLEY, D. A., SKOLIMOWSKI, I. & EDWARDS, D. I. (1979) Mechanism of action of nitroimidazole antimicrobial and antitumour radiosensitizing drugs. Effects of reduced misonidazole on DNA. *Int. J. Radiat. Biol.*, **36**, 367.

KOCH, C. J., HOWELL, R. L. & BIAGLOW, J. E. (1979) Ascorbate anion potentiates cytotoxicity of nitro-aromatic compounds under hypoxic and anoxic conditions. *Br. J. Cancer*, **39**, 321.

KOCH, C. J. & KRUUV, J. (1972) Measurement of very low oxygen tensions in unstirred liquids. *Anal Chem.*, **4**, 1258.

MASON, R. P. (1979) Free radical intermediates of foreign compounds and their toxicological significance. In *Reviews in Biochemical Toxicology.* Eds Hodgson, Bend & Philpot. Vol. 1. Amsterdam: Elsevier North-Holland Inc. p. 151.

MOORE, B. A., PALCIC, B. & SKARSGARD, L. D. (1976) Radiosensitizing and toxic effects of the 2-nitroimidazole Ro-07-0582 in hypoxic mammalian cells. *Radiat. Res.*, **67**, 459.

PALCIC, B. & SKARSGARD, L. D. (1978) Cytotoxicity of misonidazole and DNA damage in hypoxic mammalian cells. *Br. J. Cancer*, **37** (Suppl. III), 54.

PALCIC, B., SKOV, K. A. & SKARSGARD, L. D. (1980) Effects of reducing agents on misonidazole cytotoxicity. *Cancer Clin. Trials*, (in press).

STANNERS, C. P., ELICEIRI, G. L. & GREEN, H. (1971) Two types of ribosomes in mouse–hamster hybrid cells. *Nature* (*New Biol.*), **230**, 52.

STRATFORD, I. J. (1978) Split dose cytotoxic experiments with misonidazole. *Br. J. Cancer*, **38**, 130.

STRATFORD, I. J. & ADAMS, G. E. (1977) Effect of hyperthermia on differential cytotoxicity of a hypoxic cell radiosensitizer, Ro-07-0582, on mammalian cells *in vitro*. *Br. J. Cancer*, **35**, 307.

STRATFORD, I. J. & GRAY, P. (1978) Some factors affecting the specific toxicity of misonidazole towards hypoxic mammalian cells. *Br. J. Cancer*, **37** (Suppl. III), 129.

TAYLOR, Y. C. & RAUTH, A. M. (1978) Differences in the toxicity and metabolism of the 2-nitroimidazole (Ro-07-0582) in HeLa and Chinese hamster ovary cells. *Cancer Res.*, **38**, 2745.

URTASUN, R. C., BAND, P., CHAPMAN, J. D., RABIN, H. R., WILSON, A. F. & FRYER, C. G. (1977) Clinical Phase I study of the hypoxic cell radiosensitizer Ro-07-0582, a 2-nitroimidazole derivative. *Radiology*, **122**, 801.

VARGHESE, A. J., GULYAS, S. & MOHINDRA, J. K. (1976) Hypoxia dependent reduction of 1-(2-nitro-1-imidazolyl)-3-methoxy-2-propanol by Chinese hamster ovary cells and KHT tumor cells *in vitro* and *in vivo*. *Cancer Res.*, **36**, 3761.

VARGHESE, A. J. & WHITMORE, G. F. (1980) Binding of nitroreduction products of misonidazole to nucleic acids and protein. *Cancer Clin. Trials*, **3**, 43.

VARNES, M. E., BIAGLOW, J. E., KOCH, C. J. & HALL, E. J. (1980) Depletion of non-protein thiols of hypoxic cells by misonidazole and metronidazole: Implications for cytotoxicity. *Cancer Clin. Trials* (in press).

WASSERMAN, T. H., PHILLIPS, T. L., JOHNSON, R. J. & 6 others (1979) Initial United States clinical and pharmacological evaluation of misonidazole (Ro-07-0582), an hypoxic cell radiosensitizer. *Int. J. Radiat. Oncol. Biol. Phys.*, **5**, 775.

WONG, T. W., WHITMORE, G. F. & GULYAS, S. (1978) Studies on the toxicity and radiosensitizing ability of Ro-07-0582 under conditions of prolonged incubation. *Radiat. Res.*, **75**, 541.

ZANNONI, V. G. & SATO, P. H. (1975) Effects of ascorbic acid on microsomal drug metabolism. *Ann. N.Y. Acad. Sci.*, **258**, 119.